# Necrotizing and crescentic glomerulonephritis presenting with preserved renal function in patients with underlying multisystem autoimmune disease: a retrospective case series

**DOI:** 10.1093/rheumatology/keu445

**Published:** 2014-11-26

**Authors:** Stephen P. McAdoo, Anisha Tanna, Olga Randone, Frederick W. K. Tam, Ruth M. Tarzi, Jeremy B. Levy, Megan Griffith, Liz Lightstone, H. Terence Cook, Tom Cairns, Charles D. Pusey

**Affiliations:** ^1^Renal and Vascular Inflammation Section, Imperial College London, ^2^Multidisciplinary Vasculitis and Lupus Clinics, Imperial College Healthcare NHS Trust, London UK, ^3^Department of Nephrology, Ospedale Cardinal Massaia, Asti, Italy, ^4^Centre for Complement and Inflammation Research, Imperial College London, and ^5^Department of Pathology, Imperial College Healthcare NHS Trust, London UK

**Keywords:** renal, histopathology, vasculitis, anti-neutrophil cytoplasm antibody, systemic lupus erythematosus, Goodpasture’s syndrome, granulomatosis with polyangiitis, microscopic polyangiitis

## Abstract

**Objective.** Necrotizing and crescentic GN usually presents with rapidly declining renal function, often in association with multisystem autoimmune disease, with a poor outcome if left untreated. We aimed to describe the features of patients who have presented with these histopathological findings but minimal disturbance of renal function.

**Methods.** We conducted a retrospective review (1995–2011) of all adult patients with native renal biopsy–proven necrotizing or crescentic GN and normal serum creatinine (<120 μmol/l) at our centre.

**Results.** Thirty-eight patients were identified. The median creatinine at presentation was 84 μmol/l and the median proportion of glomeruli affected by necrosis or crescents was 32%. Clinicopathological diagnoses were ANCA-associated GN (74%), LN (18%), anti-GBM disease (5%) and HScP (3%). Only 18% of cases had pre-existing diagnoses of underlying multisystem autoimmune disease, although the majority (89%) had extra-renal manifestations accompanying the renal diagnosis. All patients received immunosuppression and most had good long-term renal outcomes (median duration of follow-up 50 months), although two progressed to end-stage renal disease within 3 years. We estimate that renal biopsy had an important influence on treatment decisions in 82% of cases.

**Conclusion.** Necrotizing and crescentic GN may present in patients with no or only minor disturbance of renal function. This often occurs in patients with underlying systemic autoimmune disease; early referral for biopsy may affect management and improve long-term outcomes in these cases.

## Introduction

Crescentic GN, recognized as the most severe form of glomerular inflammation, describes the histopathological finding of extra-capillary cells, both proliferating parietal epithelial cells and infiltrating leucocytes, and plasma proteins within Bowman’s space that is often associated with necrosis and fragmentation of the underlying glomerular tuft and periglomerular inflammation [1]. Classically it has been categorized based on the pattern of immunoglobulin deposition within the diseased glomerulus [2]: type I with linear IgG deposition, as seen in anti-GBM disease; type II with immune-complex deposition, seen in LN, cryoglobulinaemia and HScP; and type III with minimal or no immunoglobulin deposition (i.e. pauci-immune), associated with circulating ANCA in 80–90% of cases. It may occur in isolation as a renal-limited phenomenon, although more frequently it develops as a feature of multisystem autoimmune or rheumatic diseases [3].

The usual clinical correlate of glomerular necrosis and crescent formation is rapidly progressive GN (RPGN) [1, 2], i.e. an abrupt decline in renal function that occurs over days to weeks, in association with abnormal urinary findings of haematuria and proteinuria. Historical cohorts suggest that if left untreated, necrotizing and crescentic GN has an invariably poor outcome, with the majority of patients progressing to end-stage renal disease (ESRD) or death within months [4]. Immunosuppressive therapy has improved renal and patient survival; however, early detection and instigation of appropriate therapy is essential to prevent irreversible kidney damage and poor long-term outcomes [5].

We have observed, however, a small number of patients who have presented with preserved biochemical renal function despite demonstrating histopathological features of severe GN on renal biopsy. The aim of this study was to highlight the occurrence of these cases and to define their clinical characteristics and outcomes at our centre. For the purposes of this study we have included all cases with glomerular necrosis or crescents, regardless of the number or proportion of glomeruli involved, as we wanted to reflect the practical management of patients with these renal biopsy findings. Similarly we used a threshold serum creatinine of <120 μmol/l, regardless of the glomerular filtration rate (GFR), in order to identify those cases that were at risk of delayed diagnosis or misdiagnosis, since they did not present with the typical syndrome of RPGN. In addition, the most commonly used formula used to estimate GFR is less reliable in patients with normal or near-normal renal function, and is not validated in the context of the acute kidney injury that is usually observed with crescentic GN [6, 7].

## Methods

We retrospectively identified cases using the central renal pathology database at the Imperial College Renal and Transplant Centre, based at the Imperial College Healthcare NHS Trust, a large tertiary referral centre for nephrology that provides care to a local population of approximately 2 million in West London, UK. In accordance with the UK National Health Service Research Ethics Committee guidelines, ethics approval was not required because this work comprised retrospective data and all treatment decisions were made prior to our evaluation.

We included all adult patients with a coded diagnosis of necrotizing or crescentic GN on their first native renal biopsy between 1995 and 2011, for whom complete biopsy reports and at least 1 year follow-up were available. We excluded repeat native biopsies, paediatric cases and transplant biopsies from analysis. We included all patients with these coded diagnoses, regardless of the number or proportion of glomeruli affected, as we wanted to reflect real-world management of these biopsy findings in clinical practice. Original biopsy reports were reviewed by an experienced renal histopathologist (H.T.C.) to confirm suitability for inclusion in the study. A crescent was defined as two or more layers of proliferating cells in Bowman’s space. A normal glomerulus was defined by the absence of necrosis, crescent formation, endocapillary proliferation and segmental or global glomerulosclerosis. Glomeruli with minor mesangial or ischaemic changes only were included in this category.

To identify those cases with preserved renal function, we used a threshold of serum creatinine <120 μmol/l, the upper reference limit in our biochemistry laboratory, at the time of this diagnostic biopsy. We then reviewed case notes and laboratory records for these cases to obtain baseline demographic, clinical and biochemical data. We collected details of treatment and clinical outcomes (including renal function, proteinuria, dialysis status, inflammatory markers, autoimmune serology and mortality) at 1, 3 and 5 years and at the last follow-up.

Comparison data for the subset of patients with ANCA-associated vasculitis (AAV) in this cohort were obtained from a previously collected dataset that included 82 patients with renal biopsy–proven AAV at our centre over a similar timeframe [8]. Graphs were constructed and statistical analyses were performed using GraphPad Prism 5.0 (GraphPad Software, La Jolla, CA, USA).

## Results

### Case identification and baseline features

From a total of 3984 biopsy cases, we identified 258 with necrotizing or crescentic GN, of which 38 cases (14.7%) met our inclusion criteria of serum creatinine <120 μmol/l at presentation. Baseline patient demographics are summarized in Table 1. The median age was 57 years (range 17–78). About 68% of patients were female and the ethnicities of the cohort were in keeping with our local population.

**Table 1:** Demographics, clinicopathological diagnoses and biochemical parameters at presentation and after 1 year for the entire cohort

Variable	Value	
Demographics		
Gender, % female	68	
Age, median (range), years	57 (17–78)	
Ethnicity, *n* (%)		
Caucasian	24 (63)	
Indo-Asian	5 (13)	
Afro-Caribbean	5 (13)	
Unknown	4 (11)	

Clinicopathological diagnoses		
AAGN, *n* (%)	28 (74)	
Anti-PR3 antibody positive, *n* (% of AAV)	18 (64)	
Anti-MPO antibody positive, *n* (% of AAV)	8 (29)	
ANCA negative, *n* (% of AAV)	2 (7)	
SLE	7 (18)	
Anti-GBM disease	2 (5)	
HScP	1 (3)	

Biochemistry, median (range)	At presentation	At 1 year
Creatinine, μmol/l	84 (52–115)	82 (52–147)
eGFR, ml/min	71 (50–132)	75 (33–130)
uPCR, mg/mmol	132 (0–1700)	23 (0–274)
Albumin, g/l	29 (10–40)	38 (30–46)
Serum CRP, mg/l	17 (2–278)	3 (0–38)

AAGN: ANCA-associated GN; AAV: ANCA-associated vasculitis; eGFR: estimated glomerular filtration rate; uPCR: urinary protein:creatinine ratio.

### Histopathology

Most biopsies were diagnostically adequate, containing a median number of 14 glomeruli (range 4–33). Only seven biopsies (18%) had fewer than 10 glomeruli. Key histopathological findings for the entire cohort and according to immuno- and clinicopathological diagnosis are summarized in Table 2.

**Table 2:** Histopathological features of the index biopsy for the entire cohort and according to immuno- and clinicopathological diagnosis

	Entire cohort	Type III: pauci-immune	Type II: immune complex (IC)		Type I: linear IgG
Diagnosis	All	AAGN	SLE	HScP	GBM
Cases, *n*	38	28	7	1	2
Necrosis and crescents, median (range), %	32 (4–100)	32 (4–100)	17 (8–50)	50	36 (26–47)
Crescents, median (range), %	21 (0–100)	21 (0–100)	16 (8–71)	50	32 (17–47)
Obsolete glomeruli, median (range), %	4 (0–33)	4 (0–33)	0 (0–25)	0	9 (8–10)
Normal glomeruli, median (range), %	51 (0–92)	53 (0–83)	25 (0–92)	50	52 (43–61)
Tubular atrophy, median (range), %	10 (0–40)	10 (0–40)	5 (0–40)	10	10 (5–15)

The highest proportion of crescents was seen in type I disease, followed by type III and type II disease, similar to the ranking reported in the series where patients presented with rapidly progressive crescentic glomerulonephritis [1]. AAGN: ANCA-associated GN.

For the entire cohort, the median proportion of glomeruli affected by necrosis or crescents was 32% (range 5–100). Eight per cent of the cases were affected by segmental necrosis alone, although the majority (92%) had both necrosis and crescent formation. In 26% of biopsies, ≥50% of glomeruli were affected by necrosis or crescents. In all but one case, crescents were cellular or fibrocellular, suggesting acute pathology.

The median proportion of normal glomeruli was 51% (range 0–92). Forty-two per cent of biopsies had <50% normal glomeruli. Tubular atrophy ranged from 0% to 40% (median 10%). Overt necrotizing arteritis was reported in three cases (8%).

Table 3 summarizes the proportion of biopsies in our cohort as stratified according to the recently described histopathological classification system for ANCA-associated GN (AAGN) proposed by Berden *et al.* [9].

**Table 3:** Recently proposed histopathological classification system for ANCA-associated GN applied to our cohort

Class	ANCA-associated GN (*n* = 28), %	Entire cohort (*n* = 38), %
Focal	57	53
Crescentic	14	18
Mixed	29	29
Sclerotic	0	0

The majority of cases had focal class disease (i.e. ≥50% normal glomeruli), although a significant proportion had crescentic class (i.e. ≥50% crescentic glomeruli) or mixed class (i.e. ≤50% normal glomeruli, without predominance of crescents or global sclerosis) disease. No patients in this cohort had sclerotic class disease (i.e. ≥50% globally sclerosed glomeruli). While not validated for patients with other causes of crescentic GN, application of this classification system to our entire cohort revealed a similar distribution of histopathological classes.

### Clinical and laboratory features at presentation

The median creatinine at presentation was 84 μmol/l (range 52–115) and the median estimated GFR (eGFR) was 71 ml/min (range 50–>90). The majority of cases (79%) had an eGFR >60 ml/min.

Other biochemical features at presentation are summarized in Table 1. All patients had microscopic haematuria. All but one had detectable proteinuria [median urinary protein:creatinine ratio (uPCR) 132 mg/mmol (range 0–1700)], although in 26% this was low grade (uPCR <100 mg/mmol). Median serum albumin was 29 g/l (range 10–40). Notably, systemic inflammatory responses were generally low [median CRP 17 mg/l (range 2–278)].

**Fig. 1:**
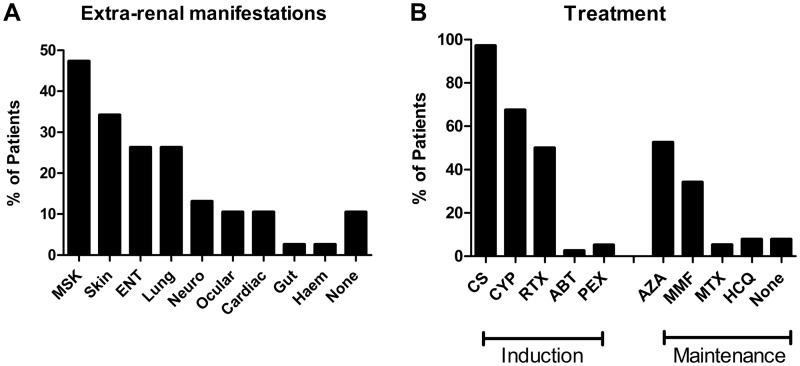
Extrarenal manifestations and immunosuppressive treatments used in this cohort (**A**) Extra-renal manifestations at the time of renal diagnosis. (**B**) Agents used for induction and maintenance immunosuppression. ABT: abatacept; Haem: haematological; MSK: musculoskeletal; Neuro: neurological; PEX, plasma exchange; RTX: rituximab.

Seven patients had pre-existing diagnoses of multisystem autoimmune disease associated with GN (four SLE and three AAV). In this group of patients, the median duration of illness prior to diagnosis of renal disease was 3 years (range 1–20). The majority of patients (89%) had extrarenal manifestations of a pre-existing or *de novo* underlying multisystem disease at the time of renal diagnosis. These extrarenal manifestations are summarized in Fig. 1A.

The most common final clinicopathological diagnosis (Table 1) was pauci-immune GN secondary to systemic AAV (74%), of which 64% were anti-proteinase 3 (PR3) antibody positive, 29% were anti-MPO antibody positive and 7% were ANCA negative. The remaining patients had SLE (seven cases), anti-GBM disease (two cases) and IgA nephropathy associated with HScP (one case). The proportion of glomeruli affected by necrosis or crescent formation according to clinicopathological diagnosis is summarized in Table 2.

### Treatment

All patients were treated with immunosuppression in accordance with local practice at the time of diagnosis (Fig. 1B). The most commonly used regimen was a combination of CS (received by 97% of cases) and either oral or i.v. CYC (68%). In 2006 we introduced rituximab as a steroid- and CYC-sparing agent in LN and AAV [10, 11], accounting for the high proportion of patients who also received this agent (50%). One patient received abatacept as part of a phase III clinical study (NCT00482066). Maintenance immunosuppressive agents included AZA (53%), MMF (34%) and MTX (5%). Three patients did not receive maintenance immunosuppression (anti-GBM disease and HScP) since they were not deemed to be at significant risk of relapsing disease. Two patients (both cases of anti-GBM disease) received plasma exchange in addition to medical therapy at the time of diagnosis.

### Outcomes

**Fig. 2:**
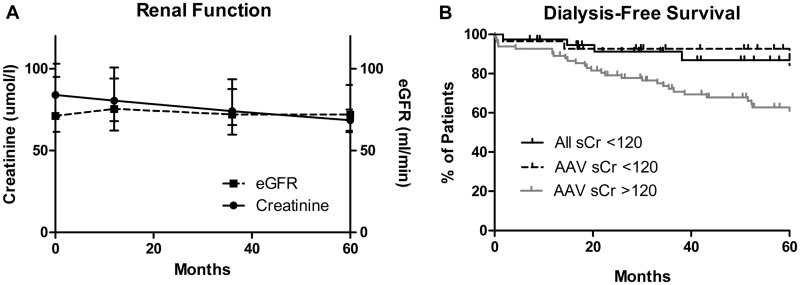
Long-term renal outcomes (**A**) Long-term renal function during the 60 month follow-up, censored for death and end-stage renal disease. Reported as median (interquartile range). (**B**) Dialysis-free survival during the 60 month follow-up for the entire cohort (all sCr <120, *n* = 38; shown in solid black). AAV cases only from this cohort (AAV sCr <120, *n* = 28; dotted plot) had significantly better rates of dialysis-free survival compared with AAV cases with serum creatinine >120 μmol/l at presentation (AAV sCr >120, *n* = 82; grey plot) over the same time period (*P* = 0.0001, log-rank test). AAV: ANCA-associated vasculitis; eGFR: estimated glomerular filtration rate; sCR: serum creatinine.

The median duration of follow-up was 50 months (range 2–181). Biochemical outcomes at 1 year are summarized in Table 1. The majority of patients had stable renal function after 1 year (median serum creatinine 82 μmol/l, median eGFR 75 ml/min; censored for death and ESRF) and beyond (Fig. 2A). Four patients died during follow-up (at 2, 15, 95 and 122 months; causes of death unknown) and two patients progressed to ESRD (at 21 and 39 months) both secondary to LN (Fig. 2B). In the AAV cohort, dialysis-free survival was significantly better in patients who presented with serum creatinine <120 μmol/l compared with those with serum creatinine >120 μmol/l over the same time period (*P* = 0.0001, log-rank test; Fig. 2B).

### Treatment decisions

We retrospectively reviewed the impact that renal biopsy results had on clinical decision making. In 37 of the 38 cases (97%), the renal biopsy was the sole means of tissue diagnosis (one patient previously had a nasal biopsy consistent with AAV). In the 31 cases (82%) who did not have extrarenal disease manifestations already requiring cytotoxic or biologic therapy—specifically, severe lung or neurological involvement—we felt that the results of the renal biopsy significantly influenced treatment decisions with regard to the choice, augmentation or duration of immunosuppressive therapy.

## Discussion

This is the largest case series of patients with biopsy-proven severe renal pathology who presented with minimal disturbance of biochemical renal function and the first series to report this finding in association with AAV. In most cases, patients had extrarenal manifestations of a multisystem autoimmune disease that facilitated early detection of asymptomatic GN, and our observations suggest that this may have improved long-term outcomes.

The most common clinicopathological diagnosis in our cohort was pauci-immune GN secondary to AAV. It has long been recognized that renal involvement is associated with poorer outcomes in AAV, with several studies reporting that renal impairment at presentation is a strong predictor of progression to ESRD and death [12–15]. The presence of proteinuria has also been associated with mortality in microscopic polyangiitis [16]. It would therefore seem logical to conclude that early detection and treatment of renal disease should improve patient outcomes, and observational data suggest that this may be the case. Holle *et al*. [17], for example, compared outcomes in three cohorts of patients (a total of 445 cases) with granulomatosis with polyangiitis diagnosed between 1966 and 2002. They found that, over four decades, as awareness of granulomatosis with polyangiitis increased and diagnostic testing improved, there was a decrease in the lag time between onset of first symptoms and diagnosis and that this was associated with lower rates of ESRD and mortality (with effects of improved treatment regimens and monitoring no doubt also contributing). However, only 50% of patients in these cohorts had renal involvement at presentation, and the nature of their renal disease was not defined. A similar, but smaller study of 181 patients with biopsy-proven AAGN diagnosed over a 30 year time period had similar findings—between 1979 and 2009, there was a decrease in serum creatinine and active lesions on kidney biopsy at the time of diagnosis, and this was associated with improved patient and renal survival [18].

No controlled studies have assessed the benefit of intense immunosuppressive treatment specifically in patients with early renal disease and preserved renal function, since the majority of trials, including those of the European Vasculitis Society, have enrolled patients with severe renal dysfunction. Only the Nonrenal Wegener’s Granulomatosis Treated Alternatively with Methotrexate (NORAM) trial [19] excluded patients with creatinine >150 μmol, and based on its findings, MTX and glucocorticoid therapy may be considered as first-line therapy for European League Against Rheumatism (EULAR) early systemic disease [20, 21]. However, the NORAM study excluded patients with significant proteinuria (uPCR >100 mg/mmol) or urinary red cell casts, a surrogate that is highly suggestive of severe GN, and thus its findings are not strictly applicable to our cohort with biopsy-proven renal disease.

Consensus opinion at our centre, based on historical series and experience of delayed referral, is that these patients are at significant risk of progression to fulminant RPGN or, alternatively, more indolent renal disease that may result in progressive glomerulosclerosis, interstitial fibrosis and tubular atrophy, particularly in patients who are anti-MPO antibody positive [22]. We therefore advocate more potent immunosuppressive therapy, as recommended for EULAR generalized stage disease, unless contraindications are present. While uncontrolled, the data reported here suggest that such treatment results in excellent long-term outcomes and are consistent with previous reports that the use of cytotoxic therapy (as opposed to CS alone) is associated with lower mortality in patients with AAV [12, 16].

We expect that future controlled studies will better define which treatments are most appropriate in this type of patient. Indeed, Berden *et al.* [9] recently proposed a histopathological classification for AAGN to enable more accurate prognostication and to facilitate stratification in clinical trials. This system describes four classes of disease: crescentic, focal, sclerotic and mixed. The first three categories are based on the predominance of crescentic, normal and globally sclerotic glomeruli in the biopsy specimen, respectively, while in the mixed disease class, none of these features is predominant. Of our cohort, 61% had focal class disease, which predicts the best renal prognosis. However, 14% had crescentic class disease and 29% mixed, for which predicted renal outcomes are reportedly worse (∼60–80% renal survival at 60 months), suggesting that our rationale for immunosuppression in this cohort was appropriate. Several validation studies of this classification system have been published, suggesting that it can reliably predict renal outcome in those with focal and sclerotic class disease, whereas crescentic and mixed class disease have variable outcomes compared with the original cohort [23]. A large international validation study is currently under way.

It could be argued that renal biopsy is unnecessary in these cases and that a diagnosis of AAGN can be assumed in patients with AAV demonstrating urinary abnormalities. Although not the subject of this study, we have identified several patients with AAV who did not have evidence of AAGN on renal biopsy; alternative pathologies included thin basement lesion, para-infectious GN and drug-induced tubulointerstitial nephritis, for which treatment significantly differs [24, 25]. Of note, Mahr *et al*. [26] recently reported an ANCA seroprevalence of almost 20% in a prospective series of patients with bacterial endocarditis, which can often mimic the clinical presentation of AAV, including the features of GN. In endocarditis, however, renal biopsy is more likely to reveal immune-complex para-infectious features rather than pauci-immune necrotizing GN, highlighting the utility of securing histology in these cases. We have previously reported on the risks of overreliance on serological testing in AAV [27], and we therefore propose that tissue diagnosis with renal biopsy should remain the gold standard for diagnosis when urinary abnormalities are present in these patients.

Seven patients (18%) in our cohort had LN. There are isolated case reports of crescentic LN presenting with normal renal function in the literature [28]; however, the finding of crescents is usually associated with significant abnormalities of renal function and poor outcome in larger series [29, 30], although these studies included only patients with >50% of glomeruli affected by crescent formation. That two of seven cases in our cohort progressed to end-stage renal failure within ∼3 years, however, highlights the important observation that patients with <50% crescentic glomeruli still have significant renal risk, despite having preserved function at presentation and receiving aggressive treatment.

Two cases of anti-GBM disease were detected in our cohort. This is widely recognized as the most aggressive form of GN, such that presentation with normal renal biochemistry is rare, though previously reported in small case series [31, 32]. Our data suggest that with appropriate treatment, including plasma exchange, these patients have a good renal prognosis.

Our study has obvious limitations: it is retrospective, uncontrolled, spans a 15 year time period and it includes a cohort of patients who have received somewhat heterogeneous treatment. However, this single-centre experience, where all patients were treated with potent, if varied, immunosuppressive regimens, illustrates several important practice points: (i) necrotizing and crescentic GN may present with preserved renal function and mild urinary abnormalities rather than the expected pattern of RPGN; (ii) this often occurs in patients with underlying multisystem disease, so non-nephrologists should be alert to the possibility of severe renal pathology, and urinalysis should be performed regularly in these patients; (iii) we found that renal biopsy findings may significantly influence treatment decisions in these patients and believe this remains the diagnostic gold standard when urinary abnormalities are present and (iv) with immunosuppressive treatment, the majority of these patients have a good renal prognosis, although a small number are at risk of progressing to ESRD.

**Rheumatology key messages**

- Crescentic GN, including ≥50% crescents, may infrequently present with minimal disturbance of renal function.
- Early detection and investigation of urinary abnormalities in patients with multisystem autoimmune disease may improve long-term outcomes.
- Renal biopsy may significantly influence treatment decisions in patients with multisystem autoimmune disease presenting with urinary abnormalities.
